# Mr. White on Arsenic in Cutaneous Complaints

**Published:** 1813-10

**Authors:** W. White

**Affiliations:** Bath


### *To the Editors of the Medical and Physical Journal.*

GENTLEMEN,

I WAS very much gratified on reading a paper in your Journal of July last, written by Dr. Kinglake, on the good effects of Arsenic in cutaneous complaints; having myself, for a great number of years, witnessed the most beneficial effects from exhibiting that medicine in the form of Fowler's Solution; especially in cases of lepra vulgaris, (according to Dr. Willan's definition,) and I do not recollect a single instance of failure in that form of disease. It has also proved highly beneficial in different species of Psoriasis, as arranged by Dr. Willan, but not so generally efficacious as in the former complaint. Although I perfectly agree with Dr. Kinglake, in regard to the superior efficacy of the medicine in question, yet my experience does not coincide with that gentleman's, as it relates to the period when the good effects resulting from its exhibition are observable; as in all the cases which have come under my care, the change has generally taken place comparatively in a short time; and the complaint has either entirely disappeared, or nearly so, before the medicine has been discontinued.

I have not administered the medicine in any case resembling Pemphigus, as that disease rarely occurs. I might also observe, that I never ventured to give so large a dose as twenty drops, having frequently found that patients have not been able to take more than six drops at a time, without producing some derangement of the stomach, although it never occasioned any bad effects to my knowledge. It is, however, not only a medicine that requires to be administered with much discrimination, but also great attention is necessary in preparing it. Some years ago I minuted several cases, in which the mineral solution had been advantageously employed; and if you think the following worth inserting in your valuable Journal, they are at your service.

I remain, Gentlemen,

*Bath,* Your most obedient Servant,

*August* 12, 1813. W. WHITE.

The

The first time I witnessed the good effects of this mineral solution in cutaneous complaints, was a case of Psoriasis diffusa, which occurred upwards of twelve years ago. A woman, about fifty years of age, came from the country for the purpose of using the Bath waters; but, in consequence of her being afflicted with asthma at the same time, I judged bathing improper, and therefore directed her to take twelve drops of the solution three times a-day. In the course of a few weeks she got perfectly free from the complaint. Some years afterwards she wrote to inform me that the disorder had returned in a slight degree, and requested to have some more of the drops, which in a short time again removed the disease.

Case II.—A lady, about fifty years of age, had been afflicted with Psoriasis inveterata for several years, and had tried a variety of medicines in vain; and also had used the Bath waters for some time, without any apparent advantage. The disorder had spread almost over the whole surface of the body. Her legs were scaly, and between the scaly patches of the cuticle there was a redness of the cutis, with a serous discharge; likewise a considerable roughness about the face and ears. Her arms were swollen; the cuticle, particularly below the elbow, was much thickened, white, with fissures. The solution was exhibited, and the dose gradually increased until she took sixteen drops three times a-day.* In the course of a few weeks the skin became nearly clean, when the lady left Bath, and took some of the medicine with her. About a year afterwards I had the pleasure of seeing her free from the complaint.

Case III.—A girl, eleven years of age, had a leprous eruption (lepra vulgaris) on her arms and legs three quarters of a year. She took Solut. Miner. gtt. viij ter indie, and used the warm bath. In six weeks she was entirely free from the eruption. After remaining well some time, the disorder returned. The same means were again employed for a few weeks, and the complaint entirely disappeared.

Case IV.—Ann Bateman, aged fourteen, had had the Psoriasis diffusa several times on her arms and hands, commencing with an itching and rising of the cuticle, which ended in fissures. Sometimes small vesicles appeared, which broke and discharged a serous fluid. She had the complaint this time about a month.

July 27.—Cap. Solut. Miner. gtt. vj ter in die.
Applic. Ung. Saturn. part affect.

She was not perfectly cured till the 10th of November.

---

* The warm bathing was continued.

Case V.—Master M——, between one and two years of age, had been afflicted with an eruption* on his face for several months, which resisted every means that had been employed, and at length spread over his arms and legs, so that almost every part of the body was affected with it. There was a serous discharge from several parts. Two drops of the solution were directed to be given twice a-day, which were increased to three drops, and to the parts that required it a cooling liniment was applied. After pursuing this course for some time, the child got perfectly well.

Case VI.—M. Young had been afflicted with the Lepra Alphos for nearly ten years. The disorder chiefly occupied the elbows and arms below, also the knees and legs. He began taking eight drops of the solution, three times a-day, on the 8th of September; and by the 19th, very little of the scaliness remained, without the assistance of warm bathing.†

Case VII.—Eliz. Sloper, aged forty, had been afflicted with Lepra Vulgaris on her arms and legs fifteen years. Some of the patches were very large, and the scales very thick. She had taken a variety of medicines without any benefit, and the disorder was increasing. Sometimes she had found a little relief from warm bathing.

Sept. 21.—Capt. Solut. Miner. gtt. viij. ter in die. Utet. Baln. tepid.

Oct. 29.—Skin almost perfectly clean. She continued well the following May.

Case VIII.—Eliz. Sheen, aged twenty-nine, had been afflicted with Lepra Vulgaris about six months; her face, arms, and legs, were nearly covered with the eruption. She took eight drops of the solution three times a-day, and in six weeks the complaint entirely disappeared.

N. B. It is now several months since, and the disorder has not returned.

Case IX.—Miss M——, about ten years of age, of a delicate habit and fair complexion, had been afflicted with Lepra Vulgaris on her arms and legs for some time; the disease had likewise affected her head, which was covered all over with dry scales of considerable thickness, assuming a honey-comb appearance. A linseed poultice was ordered to be applied to the head night and morning, which in the course of a short time removed the scales. She was directed to take six drops of the solution three times a-day, and in the course of a few weeks cured the complaint.

---

* Psoriasis diffusa.

† Nevertheless, I consider warm bathing an auxiliary, as it certainly does expedite the removal of leprous eruptions.

Case

Case X.—A gentleman, about seventy years of age, had been afflicted with Psoriasis Scrotalis for several years; and, after having consulted several of the most eminent practitioners in London, and making use of a variety of local applications by their advice, as well as undergoing a long course of alteratives in vain, was completely relieved from the complaint by the solution. The dose was gradually increased to fifteen drops three times a-day.

